# Sensory acquisition functions of the cerebellum in verbal working memory

**DOI:** 10.1007/s00429-020-02212-5

**Published:** 2021-01-22

**Authors:** Jutta Peterburs, Yu Liang, Dominic T. Cheng, John E. Desmond

**Affiliations:** 1 Department of Neurology, Division of Cognitive Neuroscience, Johns Hopkins University School of Medicine, Baltimore, MD USA; 2 Department of Biological Psychology, Heinrich-Heine-University, Institute of Experimental Psychology, Düsseldorf, Germany; 3 Department of Psychology, Auburn University, Auburn, AL USA; 4 Department of Medicine, Medical Psychology, MSH Medical School Hamburg, Hamburg, Germany

**Keywords:** Cerebellum, Cognition, Eye tracking, Working memory, Sensory acquisition

## Abstract

Several fMRI studies have shown that the superior cerebellum exhibits load-dependent activations during encoding of letters in a Sternberg verbal working memory (VWM) task. It has been hypothesized that the cerebellum regulates the acquisition of sensory data across all modalities, and thus, that VWM load activations may reflect high- vs low-load differences in sensory acquisition demands. Therefore, increased difficulty in sensory data acquisition should elicit greater activation in the cerebellum. The present fMRI study manipulated sensory acquisition in VWM by presenting visually degraded and non-degraded stimuli with high and low memory loads, thereby identifying load-dependent regions of interest in the cerebellum, and then testing if these regions showed greater activation for degraded stimuli. Results yielded partial support for the sensory acquisition hypothesis in a load-dependent region of the vermis, which showed significantly greater activation for degraded relative to non-degraded stimuli. Because eye movements did not differ for these stimulus types, and degradation-related activations were present after co-varying eye movements, this activation appears to be related to perceptual rather than oculomotor demands. In contrast to the vermis, load-sensitive regions of the cerebellar hemispheres did not show increased activation for degraded stimuli. These findings point to an overall function of association-based prediction that may underlie general cerebellar function, with perceptual prediction of stimuli from partial representations occurring in the vermis, and articulatory prediction occurring in the hemispheres.

## Introduction

A growing body of evidence is demonstrating that the cerebellum is not only involved in motor coordination and motor learning, but also in non-motor functions, such as linguistic processes (for a review see Mariën and Borgatti 2018), affective processing and emotional regulation (e.g., Lupo et al. 2018; Jeremy Schmahmann 2019; Schmahmann and Sherman 1998), executive functions (Bellebaum and Daum 2007; Bürk et al. 2003), working memory (e.g., Desmond et al. 1997; Hayter et al. 2007; Peterburs et al. 2010; Ravizza et al. 2006), and timing (Breska and Ivry 2016; Coull et al. 2011; Ivry 1997). It has been suggested that the cerebellum may sub-serve overarching functions irrespective of the domain, by involvement in more general processes of performance monitoring (Peterburs and Desmond 2016), prediction and the generation of internal models (Ito 2008; Wolpert et al. 1998), sequence detection in the context of both cognitive and motor behavior (e.g., Leggio et al. 2011; Molinari et al. 2008), and automatization (Balsters and Ramnani 2011; Ramnani 2014). Indeed, given that the cerebellum is characterized by a rather uniform neuroarchitecture, and given the existence of closed cerebro-cerebellar input–output loops (Middleton and Strick 1994; Strick et al. 2009), the idea of uniform cerebellar computations and overarching functions is very enticing. However, this idea has recently been challenged by the proposal of multiple functionality, i.e., the idea that the same underlying circuits may implement functionally distinct computations that may also differ across domains (Diedrichsen et al. 2019). Of course, this idea is not necessarily incompatible with the notion of overarching cerebellar functions, because the specific role of the cerebellar circuits could potentially change during brain development, and it is also possible that a universal cerebellar transform cannot be adequately captured in the proposed functional terms (Diedrichsen et al. 2019). In any case, the debate about universal and multiple functionality illustrates that the question of how the cerebellum contributes particularly to non-motor functions warrants further investigation.

One of the most extensively studied non-motor functions of the cerebellum is verbal working memory, i.e., the ability to temporary store and manipulate verbal information. According to Baddeley’s multi-component model (Baddeley 1992), working memory comprises a “central executive” that serves as a supervisory module controlling the flow of information to the subordinate phonological loop and visuo-spatial sketchpad which serve to temporarily rehearse and manipulate verbal and visual information, respectively. The model was later amended with an episodic buffer that links information across domains and with long-term and semantic memory (Baddeley 2000). Previous neuroimaging studies on the role of the cerebellum for verbal working memory have revealed that specific cerebellar subregions are differentially involved in encoding and maintenance/rehearsal. Activation in a superior region localized in lobule VI and Crus I during stimulus encoding that occurs in concert with activation in posterior frontal cortex has been linked to articulatory control (Chein and Fiez 2001; Chen and Desmond 2005a, b), while activation in an inferior region in lobules VIII and VIIB has been associated with phonological storage (Chen and Desmond 2005a; Desmond et al. 1997).

Importantly, neuroimaging investigations of cerebellar engagement in verbal working memory have allowed scientists to empirically test different hypotheses about the precise role of the cerebellum. For instance, a study that combined eye tracking and functional magnetic resonance imaging (fMRI) (Peterburs et al. 2016) clarified that cerebellar activations during encoding in a verbal working memory task cannot be attributed to oculomotor behavior, as had been proposed based on fronto-cerebellar connectivity patterns (Doron et al. 2010). Moreover, recent findings suggest that the cerebellum may contribute to phonological storage by predictive processes: maintenance-related inferior cerebellar activations were shown to be sensitive to repeating stimulus sequences (Peterburs et al. 2019). Another theory of overarching cerebellar function states that the cerebellum is involved in perceptual processes (a comprehensive overview of converging evidence for this notion is provided in a consensus paper, Baumann et al. 2015) and particularly in sensory acquisition, i.e., the extraction and primary processing of sensory information from the environment. Specifically, it has been proposed that the cerebellum ameliorates the efficiency with which other brain regions perform their respective functions by monitoring and adjusting sensory data acquisition (Bower 1997). This notion is supported by cerebellar activation in response to cutaneous stimulation in the absence of motor output, increased cerebellar activation when there is tactile discrimination demand, and by even more pronounced cerebellar activation when tactile discrimination is combined with motor activity (Parsons et al. 1997).

The question arises if the cerebellum is also involved in sensory acquisition outside of the tactile domain. A previous meta-analysis of functional neuroimaging studies on auditory processing revealed that specific cerebellar regions, here most notably left lateral Crus I, were activated consistently in a range of auditory tasks (Petacchi et al. 2005), which supports a general auditory function of the cerebellum that likely relates to sensory acquisition. Findings of impaired pitch discrimination in patients with cerebellar degeneration and a strong relationship between clinical ataxia scores (reflecting the extent of the cerebellar damage) and pitch discrimination deficits also corroborate this notion (Parsons et al. 2009). Moreover, activation in left Crus I was shown to scale with perceptual demands in a task that required discrimination of both visual and auditory motion signals from noise, indicating that this region may be a supramodal sensory processing region (Baumann and Mattingley 2010).

If the cerebellum is indeed involved in sensory acquisition, taxing (visual) sensory acquisition demand in a verbal working memory task should modulate the associated cerebellar activations. More specifically, load-dependent cerebellar activations during stimulus encoding in a verbal working memory task should be sensitive to a manipulation of stimulus quality. The present study tested this hypothesis using degraded and non-degraded letter stimuli in a variant of the Sternberg task (Sternberg 1966). Cognitive load-dependent regions of interest (ROIs) in the cerebellum were identified and tested for greater activation for degraded relative to non-degraded stimuli. In keeping with the notion that load-dependent cerebellar activations may reflect high- vs low-load differences in sensory acquisition demand, we hypothesized that increased difficulty in sensory data acquisition should elicit greater activations.

## Materials and methods

The present study was part of a larger investigation of cerebellar contributions to verbal working memory and presents follow-up analyses of data reported in a previous study (Peterburs et al. 2016). Importantly, this earlier study investigated whether cerebellar activation during encoding could be related to eye movements and oculomotor processing, while the present work was specifically focused on elucidating sensory acquisition functions of the cerebellum. To this end, the present study presents novel analyses of the previously unanalyzed factor stimulus quality (see task description below for details).

### Subjects

Fifteen healthy adult volunteers (10 women, 5 men) were recruited from the Baltimore community by public advertisement and on social media. Mean age was 31.6 ± 9.4 years, ages ranged 22–54 years. All subjects were native English speakers, right-handed according to self-report, and had normal or corrected-to-normal vision. Exclusion criteria included a history of neurological or psychiatric illness or head trauma, current medication affecting the central nervous system, and the following criteria pertaining to MRI scanning: claustrophobia, implanted electric or ferromagnetic devices, and pregnancy. Mean educational attainment was 18.2 ± 2 years (range: 15–21). All subjects gave written informed consent prior to participation and received monetary compensation for participation and travel expenses. The study conforms to the Declaration of Helsinki and was approved by the Johns Hopkins University School of Medicine Institutional Review Board.

### Sternberg verbal working memory task

**Fig. 1:**
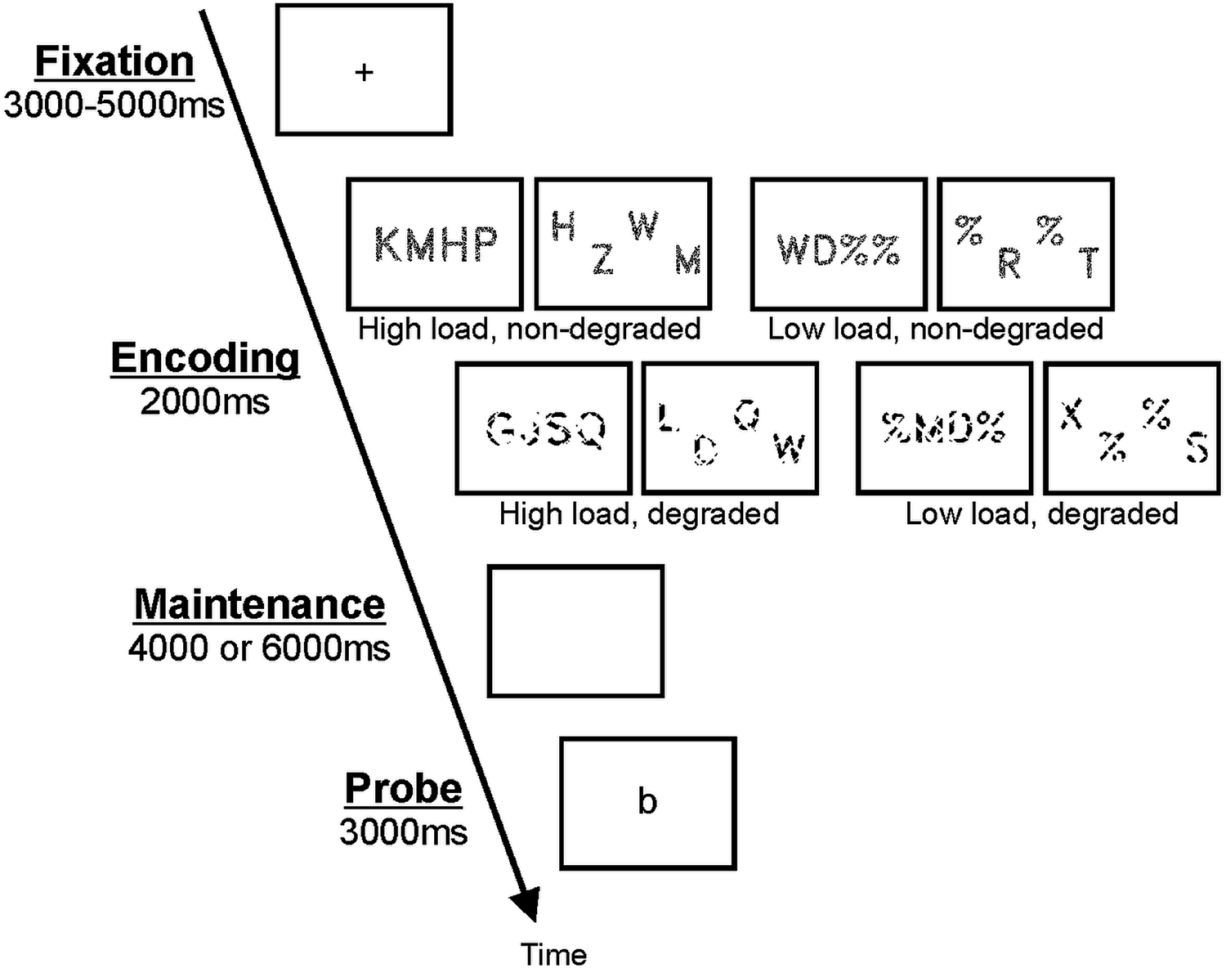
Schematic illustration of the sequence and time course of stimulus presentation in a trial of the Sternberg verbal working memory task

The experimental task was a variant of the Sternberg task (Sternberg 1966). In each trial, subjects had to keep a briefly presented set of consonants in memory by rehearsal until a probe letter was presented, and to then indicate by button press whether this probe matched one of letters in the initially presented set. The Sternberg task can be divided into discrete phases of stimulus encoding, maintenance, and retrieval which differ in terms of sensory and motor demands. The present variant of the task used normal and degraded stimulus quality (see Fig. 1) to tax sensory acquisition demand (please see below for behavioral findings from a supplementary study that confirmed increased sensory acquisition demand for the present degraded compared to the non-degraded stimuli). To tax oculomotor demand, stimulus spacing was varied in two levels (close or wide). Luminance was equated for all stimuli. To manipulate cognitive and articulatory load, two and four letter consonant sets were used. As shown in Fig. 1, percent sign (%) placeholders were displayed in unused positions in the consonant sets with only two letters. As mentioned in the previous study (Peterburs et al. 2016), the task also included overt and covert rehearsal instructions at the beginning of each trial. However, since sensory acquisition was expected to only affect the encoding phase, trials were pooled across rehearsal conditions for the present analyses. In addition, trials were also pooled across stimulus spacing conditions.

Figure 1 illustrates the sequence and time course of stimulus presentation in one trial of the Sternberg task. Each trial started with a fixation cross that was presented for 3–5 s (which implemented jitter in trial onsets for fMRI analysis), followed by a set of two or four consonants that was presented for 2 s (encoding phase). A blank screen was presented in the maintenance phase for 4 or 6 s, while subjects were rehearsing the consonants. Last, the probe was presented for 3 s, and subjects decided whether it was a “match” or “no match” by pressing one of two response buttons with their right index or middle finger. Task instructions emphasized both speed and accuracy, and accordingly, response time (RT) and accuracy were recorded for each trial. Subjects completed 10 practice trials outside the scanner prior to starting the experiment. The task consisted of three runs with 64 trials each, amounting to 192 trials in total. Stimulus quality (degraded or normal), spacing (wide or close), and cognitive load (high or low) were balanced in each run. Trial order within each run was pseudorandomized so that presentation of identical parameters was limited to three consecutive trials. Each run contained 24 unique match and 24 unique no-match trials as well as 16 trials without a probe. The latter were included to allow the hemodynamic response to fully return to baseline following the maintenance phase. In these trials, a blank screen was presented in the retrieval phase, and no response was expected. For trials with probe, positions of the target letter in the initial sequences were counterbalanced. Task completion took approximately 55 min and included short breaks between runs.

Stimulus presentation was controlled with E-Prime 2 software (Psychology Software Tools Inc., Sharpsburg, PA, USA) running on a Hewlett Packard xw4300 workstation. The visual display was rear-projected onto a screen in the MRI scanner located behind the participant and then reflected onto a mirror fixed to the head coil within the participant’s immediate line of view. Responses were recorded using two Velcro-connected fiber optic button boxes (MRA, Inc., Washington, PA).

### Behavioral test of increased sensory acquisition demand by degraded stimuli

To directly test whether degraded stimuli were producing increases in sensory processing, a behavioral pilot experiment was conducted with a sample of 16 healthy adult volunteers. Subjects completed a target detection task in each trial of which they received a lowercase target letter, followed immediately by one of the four types of visual stimuli that had been presented during the fMRI verbal working memory task described above. The subject's task was to simply indicate as rapidly as possible whether or not the uppercase version of the target letter appeared among the four items of the consonant set. The increase in reaction time (RT) needed to make this decision for degraded relative to non-degraded stimuli was used to assess the increase in sensory acquisition demand. Two stimulus lists were created such that the letters that were degraded in list 1 were non-degraded in list 2 and vice versa. Half of the subjects (*n* = 8) received list 1 and the other half received list 2. A repeated-measures analysis of variance (ANOVA) with the within-subject factors load (2 letters or 4 letters) and stimulus quality (degraded or non-degraded) revealed only a significant main effect of stimulus quality [F[1, 15]  = 60.269, *p* < 0.001], with mean RT values of 799 ± 39 ms for degraded stimuli and 716 ± 33 ms for non-degraded stimuli. These results support the notion that visual degradation significantly increased sensory processing.

### Recording of eye movements during the task

Eye movements were recorded during scanning with an Arrington Research (Scottsdale, AZ, USA) MR-compatible infrared eye tracker and the ViewPoint^®^ user interface at a sampling rate of 60 Hz. The camera was fixed to the scanner cot just outside the head coil with a flexible mount and pointed towards the left eye. Gaze point coordinates were recorded as relative position on the screen, [0, 0] reflecting the upper left and the lower right corner. Prior to the first task run, a nine-point auto-calibration procedure as implemented in ViewPoint^®^ software was applied. Here, calibration points were presented at nine equidistant locations in the stimulus display in randomized order, and subjects were instructed to foveate on each point. After all points had been presented, the recorded locations were superimposed on a 3 × 3 grid and visually checked for calibration quality. Calibration accuracy was determined based on the grid’s geometry: a rectilinear configuration indicated successful calibration. If the grid was distorted, single calibration points were re-presented to improve calibration.

Eye movement data were analyzed off-line using MATLAB (Mathworks, Natick, Massachusetts, USA). First, data were segmented according to the onset of encoding phase of each trial. Next, these segments were scanned for blink-related and other artifacts by calculating the mean and standard deviation (SD) for pupil width and by then removing data points for which pupil width fell 2 SDs above or below the mean. This procedure was applied because we assumed that pronounced pupil dilatation changes were unlikely given that lighting was consistent during MR scanning and throughout the entire duration of the experimental task. Consequently, rapid large changes in pupil width should reflect blinks (which cause the pupil to be temporarily covered by the eye lid) or scanner-related distortions which could temporarily disrupt the eye tracker’s pupil detection algorithm, resulting in incorrect gaze coordinates. Next, point-to-point absolute distances between data points were calculated and summed separately for x and y gaze point coordinates, yielding quantitative horizontal (x) and vertical (y) eye movement parameters for each trial. These parameters thus reflected the average cumulative distance the eyes “travelled” per trial/volume. Larger values reflected more eye movement, irrespective of direction or type of movement. For example, scores close to one for horizontal eye movement indicated that the eyes traveled a distance equivalent to the length of the stimulus display. Quantitative eye movement parameters were also determined for each volume acquired during scanning to generate an eye movement regressor to be included in the functional MRI analysis (see below for details).

### Analysis of working memory behavioral data

Accuracy, RT, and quantitative eye movement parameters obtained during fMRI scanning were analyzed with separate 2 × 2 repeated-measures analyses of variance (ANOVAs) with *load* (high or low) and *stimulus quality* (non-degraded or degraded) as within-subject factors. Post hoc *t* tests were performed to resolve interactions. The significance level was set to *p* < 0.05.

### MRI data acquisition and analysis

MRI data were acquired using a 3.0 T Philips Intera scanner (Philips, Eindhoven, NL). The structural MRI protocol consisted of a T1-weighted MPRAGE sequence (TR = 6.97 ms; TE = 3.3 ms; TI = 982 ms; flip angle = 8°, in-plane resolution = 0.75 mm; slice thickness = 1 mm; 170 sagittal slices; FOV = 240 mm; 1 NEX). Functional MRI data were collected using three T2*-weighted gradient echo EPI pulse sequences (TR = 1000 ms; TE = 30 ms; flip angle = 61°; in-plane resolution = 2.75 mm; slice thickness = 6 mm, gap = 1 mm; 20 oblique-axial slices; FOV = 240 mm; 1 NEX). Images were acquired in the oblique-axial plane rotated 25° clockwise with respect to the AC-PC line to optimize imaging of the cerebellum and neocortex. Application of a 1000 ms TR, jittered trial onsets, variable duration in the maintenance phase, and no-probe trials has been used previously to analyze the separate encoding, maintenance and retrieval phases of the task, and simulations confirmed that voxels responding to any combination of the three task phases could be identified (Chen and Desmond 2005b). The number of acquired volumes within each of the three runs ranged from 944–953. The start of the fMRI scan was synchronized with the start of each experimental run using E-prime software (Psychology Software Tools Inc., Sharpsburg, PA, USA).

SPM8 (Wellcome Department of Cognitive Neurology, London, UK) was used for preprocessing and statistical computations. Standard image preprocessing steps were performed: slice timing correction (reference = middle slice), motion correction, anatomical co-registration, normalization to Montreal Neurological Institute (MNI) stereotaxic space, spatial smoothing (FWHM = 5 mm). Normalization was performed based on gray and white matter segmentation as implemented in SPM. This procedure involves combining registration to standardized space and tissue classification (as gray matter, white matter, or cerebrospinal fluid) in a single generative model that includes parameters for volume intensity non-uniformity (Ashburner and Friston 2005). This procedure has been shown to yield cerebellar output that closely corresponds to that of SUIT (as opposed to the SPM default non-linear normalization method, see Diedrichsen et al. 2009).

### First-level fMRI analysis

Individual statistical maps were computed for each subject using the general linear model approach as implemented in SPM8, with high-pass filtering of 128 s. Since the present investigation focused on whether sensory acquisition processes could be responsible for working memory load-dependent activations, fMRI analysis was focused on the encoding phase of the task, but the other task phases (maintenance and retrieval) were nevertheless also modelled in the GLM, as were the high vs low load and degraded vs non-degraded trial types. Thus, because there were two load conditions, two stimulus quality conditions, and trials were divided into three phases, each scan had a total of 12 task-related regressors for first-level analysis. The duration of these regressors was 2 s for the encoding phase, 4 or 6 s for the maintenance phase, and 3 s for the retrieval phase. For no-probe trials, the retrieval phase was omitted. For fMRI analyses, these regressors were convolved with the SPM canonical hemodynamic response function. Only correct trials were modelled, given that overall performance accuracy was very high (94.5%). Additional regressors of no interest for each scan included motion parameters computed from motion correction and artifacts identified with Artifact Detection Tools (ART; https://www.nitrc.org/projects/artifact_detect), a toolbox for fMRI post-processing optimized for artifact removal. Because each subject completed three scans total for the fMRI session, the GLM for first-stage analysis included all three scans, and contrasts were pooled across these scans. Contrasts of interest that were included in the second-stage (random effects) analysis were specific to the encoding phase and included working memory load (high–low), stimulus quality (degraded–non-degraded), and the load x stimulus quality interaction. These were each implemented as *t* test contrasts.

### Second-level fMRI analysis

Second-level (random effects) analyses were performed by computing (from the first level analysis) one contrast volume per subject for the working memory load, stimulus quality, and load × quality interaction contrasts, and then using these volumes to calculate one-sample *t* test values at every voxel (testing the null hypothesis that the contrast value was 0). Because behavioral analyses indicated that eye movements’ differences during the encoding phases of degraded vs non-degraded stimulus trials approached significance, in the fMRI second-level analysis of the degraded–non-degraded contrast, trial-wise eye movement parameters were included as a co-variate. The analysis strategy at group level was to first identify cerebellar activations in left and right hemispheral lobule VI/Crus I and vermis, which have been shown to exhibit a significant working memory load effect (high > low) in previous studies (Chen and Desmond 2005a, b; Desmond et al. 1997). We identified these clusters in the designated regions using a voxel-wise significance level of *p* < 0.005. These clusters were used to create activation-defined regions of interest (ROIs), consisting of all contiguous voxels that reached the *p* value threshold. Further analyses of stimulus quality were conducted on these ROIs using a small volume correction (voxel-wise *p* < 0.005 threshold, peak FWE-corrected *p* < 0.05). In addition, we performed FWE-corrected (voxel-wise *p* < 0.005 threshold, peak FWE-corrected *p* < 0.05) whole brain analysis of the stimulus quality effect (degraded > non-degraded), and subsequently analyzed the load effect (high > low) in brain regions that showed greater activation for degraded relative to non-degraded stimuli. To further examine if additional regions of the brain were responsive to both sensory acquisition demand and working memory load, we performed an additional conjunction analysis in which voxels that were significant for both load and stimulus quality contrasts (at a voxel-wise *p* < 0.05 to maximize potential regions of overlap) were identified. We then examined peak activation within these clusters to identify all conjunction regions in which peak activation within the cluster exceeded *p* < 0.005 for both the load contrast and for the stimulus quality contrast.

MNI coordinates of activation peaks were transformed into the coordinate system of the Talairach and Tourneaux stereotaxic atlas (Talairach et al. 1988) using the MNI to Talairach transformation described by Lancaster et al. (1997) to make anatomical determinations of the neocortical activations (which were verified by manual inspection). However, MNI coordinates are reported in tables and figures. For the cerebellum, MNI coordinates were referenced with the SUIT atlas (Diedrichsen et al. 2009).

## Results

### Behavioral data

**Fig. 2:**
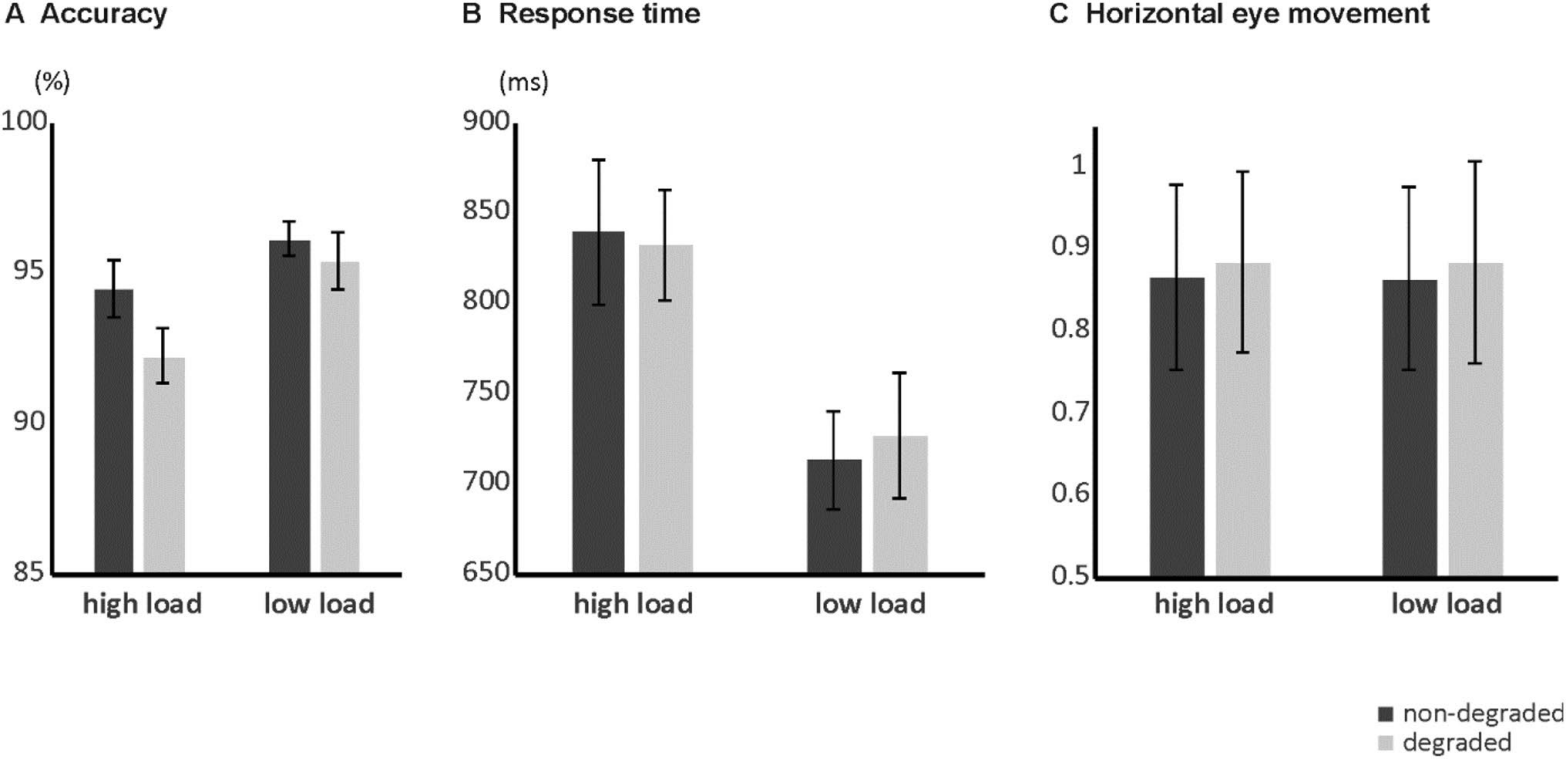
Working memory behavioral results. Mean percentage of correct response **a**, mean RTs **b** and mean eye movement parameters **c** according to load and stimulus quality

Mean percentages of correct responses according to load and stimulus quality are provided in Fig. 2a. For accuracy, the ANOVA yielded a significant main effect of load (F[1, 14] = 7.492, *p* = 0.016), indicating that response accuracy was higher for trials with low (mean = 95.74%, SE = 0.57) relative to high cognitive load (mean = 93.33%, SE = 0.69). Moreover, there was a trend for a main effect of stimulus quality F[1, 14] = 3.276, *p* = 0.092), reflecting slightly better performance for non-degraded (mean = 95.27%, SE = 0.62) compared to degraded stimuli (mean = 93.80%, SE = 0.60). The load x stimulus quality interaction failed to reach significance (*p* = 0.395).

Mean RTs for correct responses according to load and stimulus quality are provided in Fig. 2b. The ANOVA revealed a significant main effect of load (F[1, 14] = 60.188, *p* < 0.001), with longer RTs when cognitive load was high (mean = 836 ms, SE = 34) compared to low (mean = 720 ms, SE = 30). No other effects reached statistical significance (all *p*’s > 0.461).

Eye movement means according to load and stimulus quality are provided in Fig. 2c. Note that scores close to one for horizontal eye movement (as observed in the present task) suggest that the eyes traveled a distance equivalent to the length of the whole stimulus display which ranged from positional coordinate [0] for the left side to [1] for the right side. Eye movement analysis did not reveal any significant main effects or interactions (all *p*’s > 0.274), although there was a trend for a significant main effect of stimulus quality (F[1, 14] = 2.974, *p* = 0.107), pointing towards more movement for degraded (mean = 0.89, SE = 0.12) relative to non-degraded stimuli (mean = 0.86, SE = 0.11).

### Imaging data

**Fig. 3:**
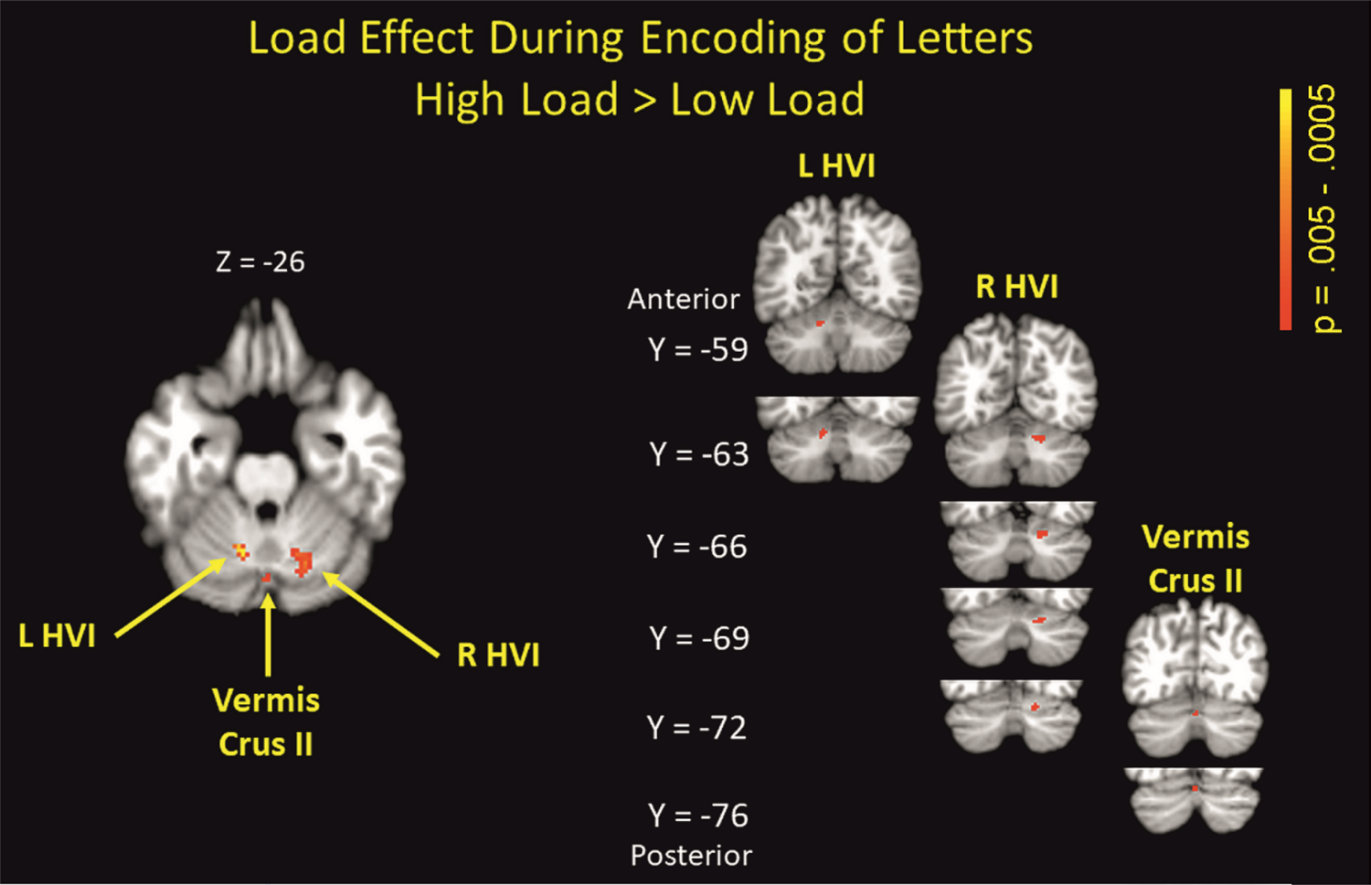
Positive cerebellar activations for high vs low cognitive load (high > low). *p* < 0.005 uncorrected. *LHVI* left hemispheral lobule VI, *RHVI* right hemispheral lobule VI

**Fig. 4:**
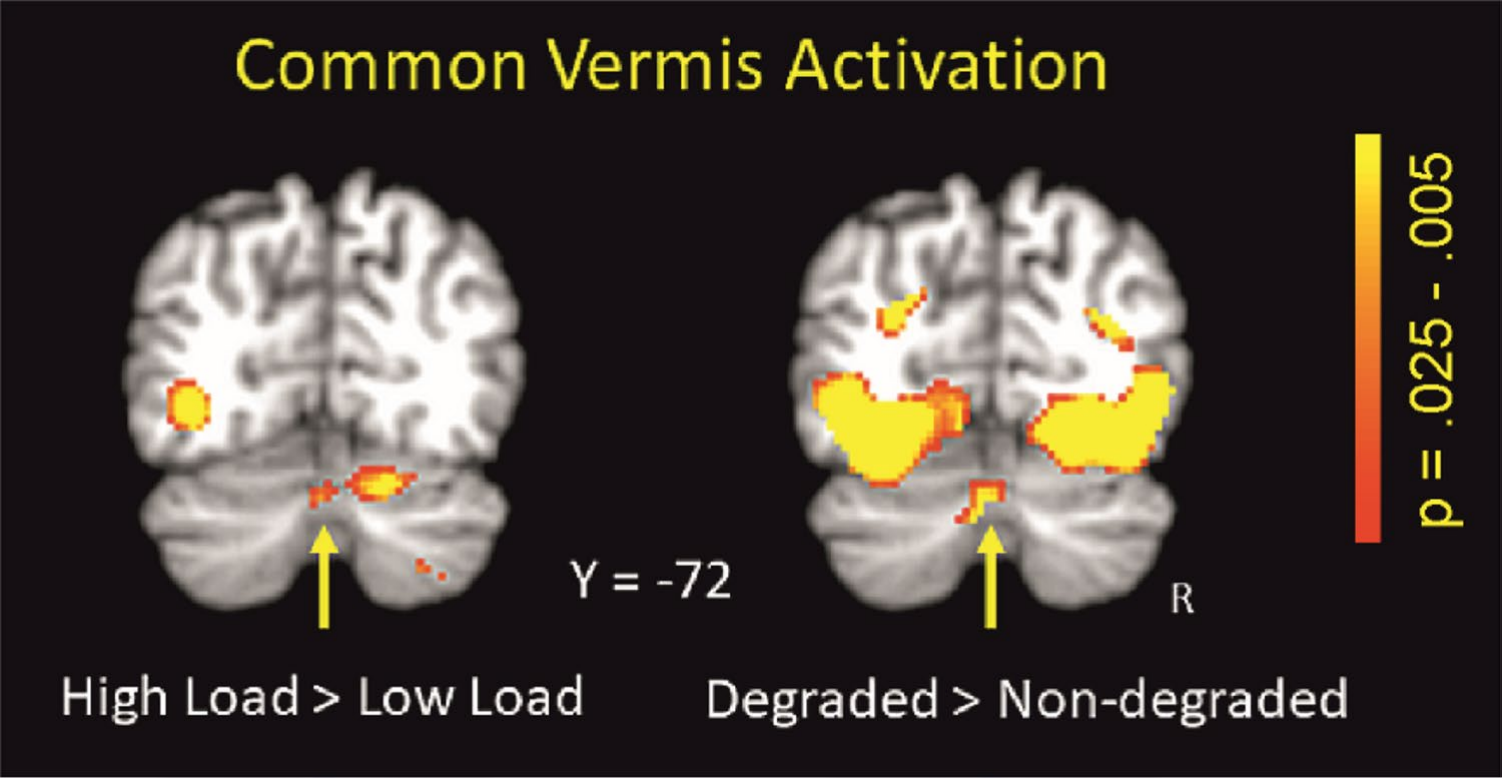
Load effect (high > low; left panel) and stimulus quality effect (degraded > non-degraded; right panel) in Vermis Crus II (small volume corrected, *p* < 0.005)

BOLD signal changes for the load effect were observed in mainly three cerebellar regions. Left hemispheral lobule VI, right hemispheral lobule VI, and vermis Crus II (see Fig. 3 and Table 1) all showed greater activation for high compared to low load. Subsequent analysis of stimulus quality effects in these load-dependent activation-defined cerebellar ROIs yielded significantly increased activation for degraded relative to non-degraded stimuli only in vermis Crus II (see Fig. 4), but no interaction of load and stimulus quality was observed.

**Table 1:** MNI coordinates of activation maxima for the cerebellar load effect (voxel-wise *p* < 0.005 threshold uncorr), the stimulus quality effect in load-dependent cerebellar ROIs (small volume corrected, voxel-wise *p* < 0.005 threshold, peak FWE-corrected *p* < 0.05), the extra-cerebellar stimulus quality effect (whole brain, voxel-wise *p* < 0.005 threshold, peak FWE-corrected, *p* < 0.05), and for the load-effect in stimulus-quality dependent extra-cerebellar ROIs (small volume corrected, voxel-wise *p* < 0.005 threshold, peak FWE-corrected *p* < 0.05)

Brain region	X	Y	Z	SPM (Z)	Size (mm^3^)
Significant cerebellar activations for high > low load during encoding					
Left hemispheral lobule VI	− 12	− 62	− 26	3.30	1006
Right hemispheral lobule VI	16	− 64	− 28	2.95	2330
Vermis crus II	0	− 74	− 28	2.73	371
Right hemispheral lobule VIIb	32	− 70	− 52	2.90	53
Significant activation for degraded > non-degraded in load-defined cerebellar ROIs					
Vermis crus II	− 2	− 74	− 28	2.60	53
Significant extra-cerebellar activations for degraded > non-degraded during encoding					
Left fusiform gyrus	− 36	− 72	− 12	5.82	69,295
Right inferior occipital gyrus	40	− 74	− 4	4.94	80,148
Significant activation for high > low load in stimulus quality- defined extra-cerebellar ROIs					
Left fusiform gyrus	− 36	− 74	− 4	3.62	4129

**Fig. 5:**
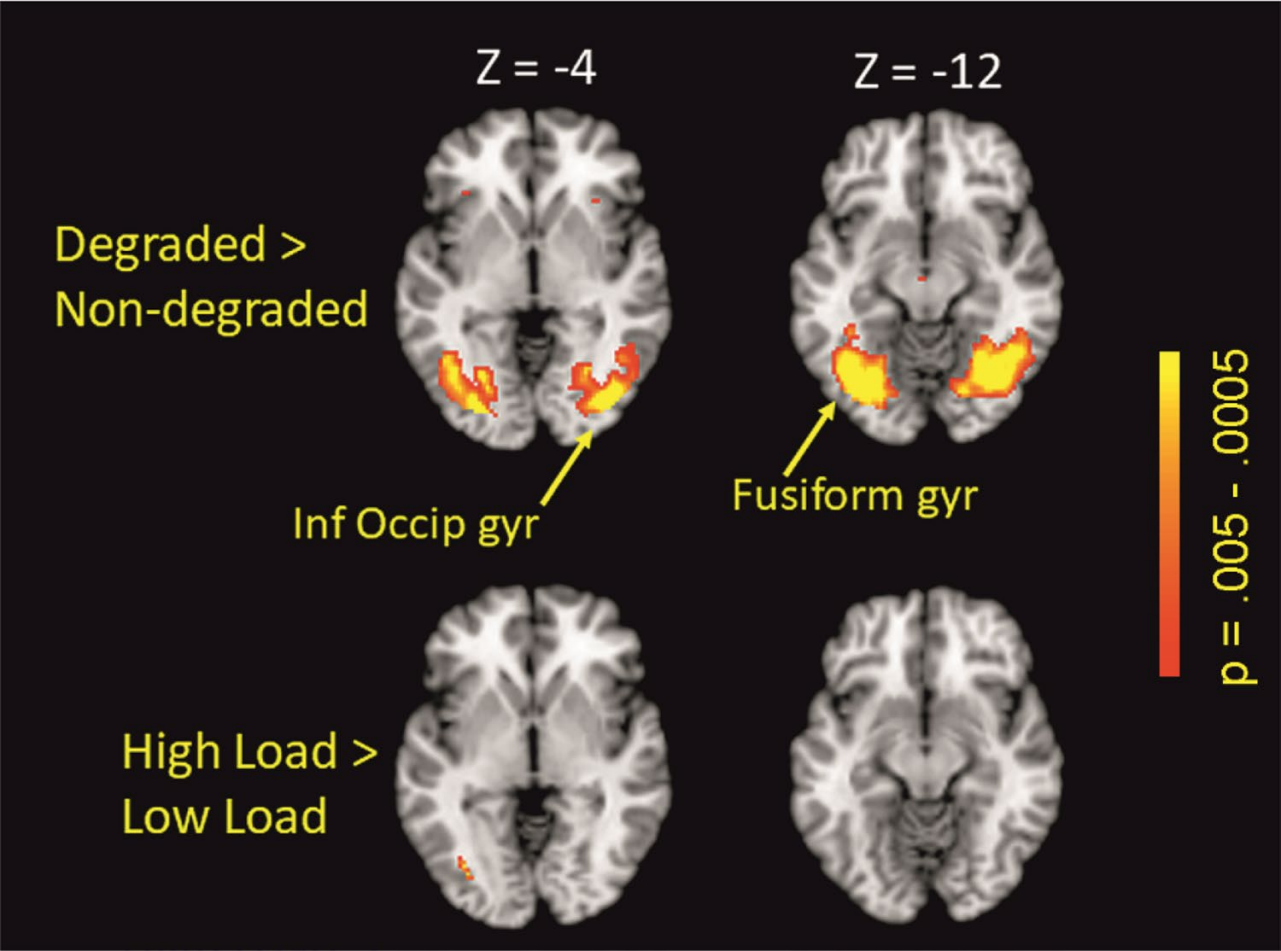
Stimulus quality effect (degraded > non-degraded; whole brain, FWE-corrected, *p* < 0.05) in left fusiform gyrus and right inferior occipital gyrus (upper panel), and load effect (high > low) in a small cluster in left fusiform gyrus (lower panel). *Inf Occip Gyr* inferior occipital gyrus, *Fusiform gyr* fusiform gyrus

Additional analyses of extra-cerebellar effects of stimulus quality were investigated in a whole brain analysis. Consistent with the behavioral results found from our initial test of increased sensory acquisition demand by degraded stimuli (i.e., increased RT for degraded relative to non-degraded stimuli), greater activation for degraded vs non-degraded stimuli was found in a cluster in the left fusiform gyrus and a cluster in right inferior occipital gyrus, further indicating that the degradation manipulation had indeed increased sensory acquisition demand (see Fig. 5 and Table 1). Using the respective activation-defined ROIs in left fusiform gyrus and right inferior occipital gyrus as ROIs, subsequent analyses of load identified only a small cluster in left fusiform gyrus that showed greater activation for high relative to low load; there were no suprathreshold clusters in the right inferior occipital region. However, fusiform activations were much more extensive for the stimulus quality effect than for the load effect.

The conjunction analysis of working memory load and stimulus quality revealed only three regions in which peak activity in the conjunction region reached *p* < 0.005 for both contrasts, the left fusiform gyrus region shown in Fig. 5, the Vermis Crus II region shown in Figs. 3, 4, and a 900 mm^3^ region in the left middle frontal gyrus (Brodmann Area 6) located at MNI coordinates -34, 0, 46 using the load contrast peak, and at coordinates -36, 2, 46 using the stimulus quality contrast peak.

## Discussion

The present study investigated whether cerebellar activations in a verbal working memory task were modulated by perceptual demands. In line with the a priori hypothesis, and in support of sensory acquisition as a general function of the cerebellum, load-dependent activations in vermis Crus II scaled with sensory acquisition demand and were increased for degraded relative to non-degraded stimuli. In contrast, activations in bilateral hemispheral lobule VI, which were also increased for high compared to low cognitive load, were unaffected by stimulus quality. In general, this result pattern suggests that the cerebellar hemispheres are involved in articulatory processing and representation, while perceptual processes related to the prediction of visual stimuli from partial representations occur in the vermis. Of note, cerebellar activations in the present study cannot be attributed to motor effects since eye movements were used as covariates in the analysis.

The remarkably uniform neuroarchitecture of the cerebellum and the identification of distinct reciprocal loops that connect different cerebellar regions to distinct motor and cognitive regions in the neocortex (Middleton and Strick 1994; Strick et al. 2009) have inspired a search for overarching, domain-independent functions of the cerebellum. To date, a range of such functions have been proposed, e.g., prediction and internal models (Ito 2008; Wolpert et al. 1998), general functions of performance monitoring (Peterburs and Desmond 2016), automatization (Balsters and Ramnani 2011), timing (Ivry 1997), and sensory acquisition (Bower 1997). The present study was aimed to further investigate the notion that the cerebellum is involved in perceptual processes (Baumann et al. 2015), and here specifically sensory acquisition. A Sternberg verbal working memory task was used because this type of task has been shown to reliably produce robust cerebellar activation patterns that inform about distinct functional roles of specific cerebellar subregions (e.g., Chen and Desmond 2005a, b; Desmond et al. 1997). Since sensory acquisition processes are most critical during initial stimulus encoding, data analysis in the present study focused on the encoding phase of the Sternberg task. In accordance with previous findings (Chein and Fiez 2001; Chen and Desmond 2005a, b; Kirschen et al. 2005), cognitive load, i.e., the number of letters to be encoded into an articulatory code, modulated activations bilaterally in the cerebellar hemispheres in lobule VI. In previous studies, activations in right lobule VI occurred concordant with activations in left inferior frontal cortex, linking them to articulatory representations (Chein and Fiez 2001; Chen and Desmond 2005b).

Interestingly, in the present study, a smaller region in vermis Crus II was also sensitive to the load effect, with increased activation for high relative to low load. Importantly, activations in this region were also modulated by stimulus quality: in line with our a priori hypothesis, degraded stimuli that required more sensory processing than non-degraded stimuli, as confirmed by increased RTs in a separate behavioral test of the stimuli, were associated with increased activation. These effects of load and stimulus quality on activity in vermis Crus II are particularly interesting, because this area was shown to both receive projections from area 46 (middle frontal gyrus/dorsolateral prefrontal cortex) and project back to this area (rather than to M1 or oculomotor regions) in a primate neurophysiology study using neurotropic virus tracing in macaques (Kelly and Strick 2003). Human area 46 and macaque area 46 were also shown to closely match in terms of cortical functional connectivity in a later study (Neubert et al. 2014), corroborating the notion of functional correspondence. Importantly, area 46 has been implicated in working memory for different types of stimuli in a large number of studies (for a meta-analysis of fMRI studies, see Owen et al. 2005), and the present findings of memory load and stimulus quality effects in a cerebellar region densely connected to area 46 are well in line with this.

Apart from vermis Crus II, increased activation for degraded relative to non-degraded stimuli was also found in the visual cortex in right inferior occipital gyrus and left fusiform gyrus. The left fusiform gyrus also showed load-dependent activation differences, albeit to a much lesser degree than the modulation by stimulus quality. Overall, this result pattern is consistent with increased sensory and attentional demand for degraded compared to non-degraded stimuli. Of note, adjacent vermal regions of lobule VI have been linked to processing of visual motion cues (Baumann and Mattingley 2010) as well as biological motion based on point-light animations (e.g., Ferrari et al. 2019), while vermis lobule VII/Crus II has been associated with processing of emotionally salient stimuli (for a review, see Stoodley and Schmahmann 2009).

Interestingly, conjunction analysis identified (in addition to vermis Crus II and left fusiform gyrus) a small region in left middle frontal gyrus (BA 6) that was also responsive to manipulations of both load and stimulus quality. Prior work has linked BA 6 to executive processes during maintenance and thus monitoring and manipulation demands (e.g., Wager and Smith 2003; Nee et al. 2013). Previous studies from our lab (Desmond et al. 1997; Chen and Desmond 2005a, b) have linked superior cerebellar activation to posterior frontal activations associated with the articulatory control system of the Baddeley model of verbal working memory (Baddeley 1992, 2000). The present results extend these findings and suggest that a BA6-vermis crus II circuit could sub-serve both working memory load and sensory acquisition demands.

In general, increased activations for decreased signal strength (and hence increased sensory demand) have previously been reported in a task in which visual and auditory motion had to be distinguished from noise (Baumann and Mattingley 2010). It has been proposed that cerebellar input is used to ameliorate processing/signal acquisition in sensory regions, e.g., by facilitating computational efficiency and providing sensory data control (Bower 1997). This notion was based on early fMRI investigations in the tactile domain that disentangled mere finger movement control from the use of the fingers as sensory devices (Gao et al. 1996). Specifically, it was shown that activation in the dentate nucleus, the sole output node for the cerebellar hemispheres, was greater for sensory discrimination tasks with and without movement than for a task involving finger movement not associated with tactile sensory discrimination. In the auditory domain, a meta-analysis of 15 studies showed consistent cerebellar activations across a range of different simple auditory tasks that could not be attributed to attentional demand (Petacchi et al. 2005), thus supporting a role for the cerebellum in auditory sensory processing that is consistent with the sensory acquisition hypothesis. In line with this, patients with cerebellar degenerative disease relative to healthy control subjects show impaired pitch discrimination despite normal hearing thresholds and similar performance in control tasks involving sustained attention and verbal auditory working memory (Parsons et al. 2009). Aside from these empirical findings, a role for the cerebellum in sensory acquisition is also supported by evolutionary considerations. Paulin (1993) provided several examples of species in which lobular expansion in the cerebellum seems to track sensory demands better than motor demands. For example, echolocating microbats and whales (which have different motor requirements, i.e., bats use distal muscles for movement whereas whales use axial muscles) show similar expansion in vermal lobule VIII that is not seen in non-echolocating bats and whales. Paulin therefore proposed that the cerebellum may serve as a tracking system that is important for the control and coordination of movements which requires that moving objects as well as own movements are tracked and that sensory consequences of movements are analyzed appropriately. Importantly, the cerebellar association with passive echolocation and passive electrolocation is analogous to that found for active sensory systems, indicating that cerebellar sensory acquisition functions are not dependent on movement (Paulin 1993). Along these lines, in the present working memory task with visual stimuli, it is conceivable that cerebellar input may be used to improve the prediction of stimulus identity when stimuli are degraded. This would be consistent with the notion of cerebellar internal models and predictive functions (Ito 2008; Wolpert et al. 1998).

The cerebellar role in sensory acquisition yields interesting implications for processes of learning and expertise-building. A very recent study revealed a pattern of reduced cerebellar activation that accompanied higher sensory cortical activity in experienced archery athletes. In contrast, in non-athletic control participants, visual activations were found to co-occur with extensive cerebellar activation (Lo et al. 2019). This result pattern appears to suggest that cerebellar sensory data control may be needed less when expertise is high, i.e., when sensory processing is already optimized. Future studies should investigate if this also applies to other non-motor functions.

To conclude, the present findings provide partial support for the sensory acquisition hypothesis and point to an overall function of association-based prediction that may underlie general cerebellar function, with perceptual prediction of stimuli from partial representations occurring in vermal and articulatory prediction occurring in hemispheral regions. Such a function is well in line with performance monitoring accounts of cerebellar function and with the generation of internal models for both motor and non-motor functions. On the other hand, the idea of an overarching cerebellar function has been challenged by the proposal of cerebellar multiple functionality (Diedrichsen et al. 2019). Along these lines, the regional non-uniformity of function observed in the present study (i.e., load but not sensory acquisition sensitivity of the cerebellar hemispheres) could also be viewed as evidence supporting cerebellar multiple functionality. More research is therefore needed to unravel the specific nature of the cerebellar involvement in well-defined task domains.
